# Ileal Obstruction: A Rare Complication of Percutaneous Endoscopic Gastrostomy Tube Insertion

**DOI:** 10.7759/cureus.114389

**Published:** 2026-08-11

**Authors:** Fedaa Housen, Bashar Almasri

**Affiliations:** 1 Gastroenterology and Hepatology, Ibrahim Bin Hamad Obaidullah Hospital, Ras Al Khaimah, ARE

**Keywords:** enteral nutrition, ileal perforation, peg tube complication, percutaneous endoscopic gastrostomy, small bowel obstruction

## Abstract

Percutaneous endoscopic gastrostomy (PEG) is a widely used and generally safe method for establishing long-term enteral feeding. Bowel obstruction caused directly by the gastrostomy tube is a rare and easily overlooked complication, as the initial symptoms closely resemble ordinary feed intolerance. We report the case of a 55-year-old bedridden man with primary antiphospholipid syndrome and recurrent ischemic strokes who underwent uncomplicated PEG tube insertion for long-term nutritional support following aspiration pneumonia. Enteral feeding commenced the following day without difficulty. Ten days later, he developed recurrent vomiting and progressive feed intolerance. Plain CT of the abdomen demonstrated dilated small bowel loops consistent with obstruction. Subsequent contrast-enhanced CT identified a high-grade transition point in the ileum immediately adjacent to the PEG insertion site, with surrounding fibrotic tissue change. At surgical exploration using a hybrid laparoscopic-endoscopic approach, the gastrostomy tube was found to have eroded through an adherent loop of ileum, creating two full-thickness perforation sites in the bowel wall. The ileal defects were repaired primarily, the old tube retrieved endoscopically, and a new gastrostomy fashioned in the same operative sitting. The patient recovered without further vomiting, tolerated feeds through the revised tube, and follow-up contrast CT confirmed complete resolution of the obstruction. This case underscores that PEG-related bowel injury, while rare, should be considered in any patient who develops feed intolerance or vomiting following gastrostomy tube placement and does not settle quickly. Contrast-enhanced CT and prompt multidisciplinary surgical involvement are essential for timely diagnosis and a good outcome.

## Introduction

Percutaneous endoscopic gastrostomy (PEG) is the preferred technique for long-term enteral access in patients who cannot maintain adequate oral intake, most commonly those with neurological dysphagia following stroke [1]. The pull (Ponsky-Gauderer) technique is most widely used and is considered low-risk in experienced hands [1-9].

Most post-procedural problems are minor: peristomal wound infection, leakage around the tube site, and inadvertent dislodgement [2]. Serious complications, including gastric or colonic perforation [3] and buried bumper syndrome [4,5], are infrequent. Small bowel obstruction directly caused by the tube is rare and has been described most often when a bowel loop is interposed between the stomach and abdominal wall at the time of insertion, the so-called sandwich phenomenon, with symptoms sometimes not appearing until days to weeks later [6-11].

Preexisting conditions that promote intra-abdominal adhesion formation, including prior abdominal surgery, chronic peritoneal inflammation, prolonged recumbency, or repeated abdominal infections, may alter the normal anatomical relationships between the stomach and adjacent bowel loops, predisposing susceptible patients to tube-related visceral injury even after a technically uncomplicated procedure [9,10]. Although our patient had no prior abdominal surgery, his prolonged bedridden state and recurrent systemic infections may represent plausible contributors to the peritoneal micro-inflammatory state that likely facilitated ileal adhesion to the gastric wall in this case.

We report a case of ileal obstruction developing 10 days after an uncomplicated PEG insertion, caused by the gastrostomy tube eroding through a loop of ileum adherent to the anterior gastric wall. This report was prepared in accordance with the CAse Report (CARE) and Surgical CAse Report (SCARE) 2020 guidelines [12,13].

## Case presentation

A 55-year-old bedridden man with type 2 diabetes mellitus, hypertension, dyslipidemia, and primary antiphospholipid syndrome was admitted in 2025 with fever, confusion, and poor oral intake of several days’ duration, with a single episode of vomiting. He had sustained five ischemic strokes since 2019, leaving him permanently bedridden with bilateral limb contractures, pressure ulcers, and post-stroke epilepsy. His regular medications included apixaban 2.5 mg twice daily. He had no prior history of abdominal or pelvic surgery, and no known intra-abdominal pathology had been documented before this admission.

Urine culture on admission yielded multidrug-resistant *Escherichia coli*, sensitive only to gentamicin and amikacin (Table 1). During the admission, he was treated for aspiration pneumonia, a UTI, and concurrent COVID-19. Approximately two and a half weeks into his admission, he developed acute critical ischemia of the left lower limb. A vascular surgery opinion at a tertiary center concluded he was not a candidate for thrombectomy, and thrombolysis was contraindicated; he was managed conservatively with therapeutic-dose enoxaparin with a plan to transition to apixaban 5 mg twice daily once stable. Bullae at the base of the left great toe were incised and drained at the bedside, with IV clindamycin coverage. Given his prolonged inability to maintain oral intake and the irreversible nature of his neurological impairment, the multidisciplinary team agreed to insert a PEG tube for long-term enteral nutritional support.

**Table 1:** Key laboratory parameters at clinically significant time points during the patient’s admission.

Parameter	Admission day	Day 10 (obstruction identified)	Day 21 (preoperative)	Day 26 (postoperative follow-up)
WBC (× 10⁹/L)	13	12.5	13.8	7.5
Neutrophils (× 10⁹/L)	10	10	11.2	5
CRP (mg/L)	90	55	82	42
Hemoglobin (g/dL)	10	10.5	9.8	9
Albumin (g/L)	25	28	20	20
Creatinine (μmol/L)	78	90	105	90
Estimated glomerular filtration rate (mL/min/1.73m²)	87	75	64	75
Sodium (mmol/L)	144	145	145	140
Potassium (mmol/L)	3.4	3.8	3.9	3.5
International normalized ratio	1	1.2	1.2	1.3
Platelet count (× 10⁹/L)	380	233	250	243
Blood culture	Negative	N/A	N/A	N/A
Urine culture	Multidrug-resistant *Escherichia coli*>10⁵ cfu/mL	N/A	N/A	N/A

PEG tube insertion

The tube was placed using the standard pull (Ponsky) technique [1]. Upper gastrointestinal endoscopy was unremarkable. The stomach was insufflated, and transillumination confirmed the planned puncture site. A 20 Fr gastrostomy tube was inserted in a single attempt, secured at 20 cm, with intragastric position confirmed endoscopically. No complications occurred. Enteral feeding was commenced the following day at 10 mL/hour with planned advancement toward the goal rate, alongside ongoing IV fluids.

Development of bowel obstruction

Ten days after PEG placement, the patient developed recurrent vomiting and was unable to tolerate feeds. Plain CT of the abdomen demonstrated markedly dilated small bowel loops with multiple air-fluid levels and a collapsed large bowel, consistent with small bowel obstruction. The gastric tube was noted in its expected position; there was no pneumoperitoneum or intraperitoneal fluid. Incidentally, the left kidney was small, and a nonobstructing right renal calculus was noted without back-pressure change.

**Figure 1:**
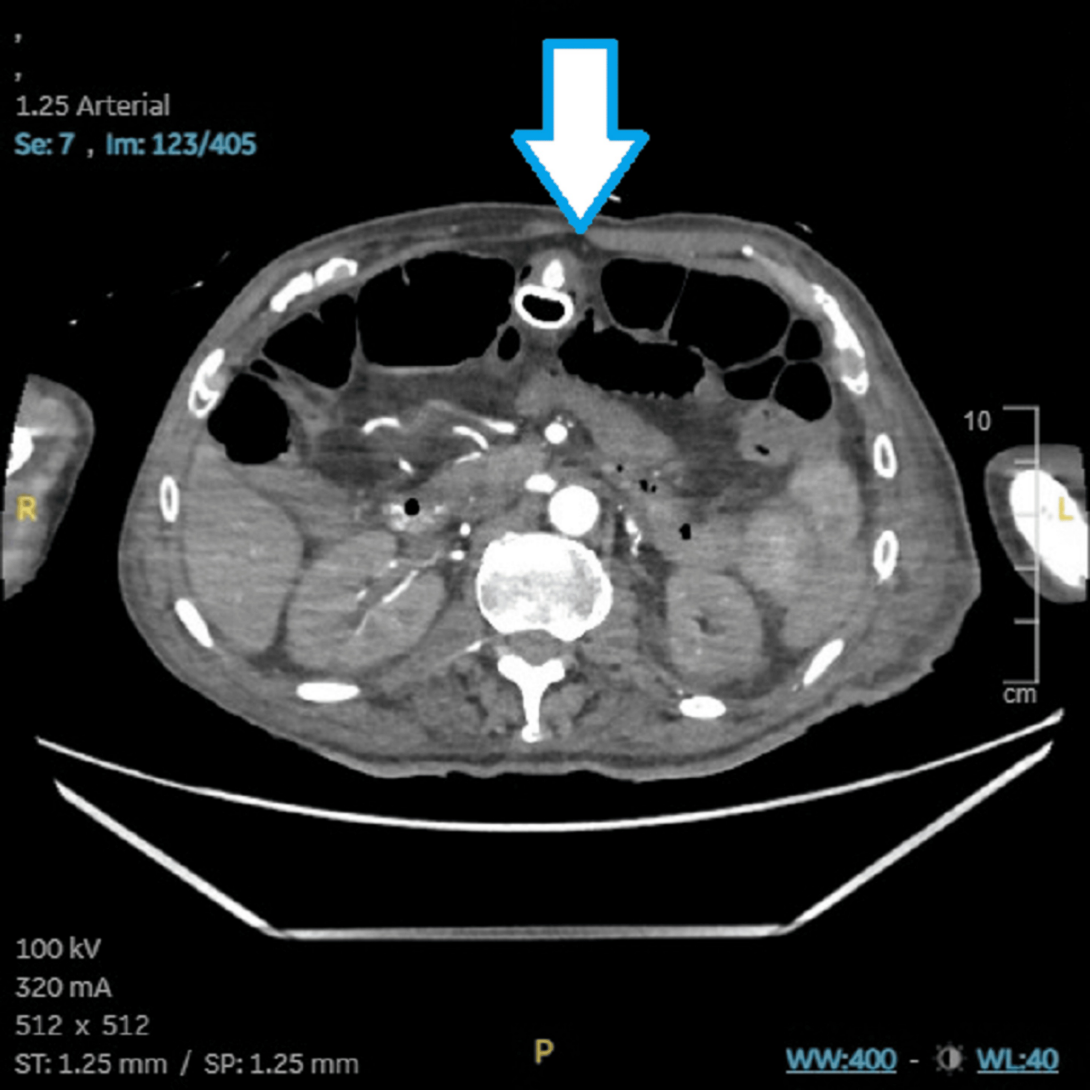
Contrast-enhanced axial CT of the abdomen showing markedly dilated, fluid-filled small bowel loops proximal to the obstructive transition point in the ileum, located immediately adjacent to the PEG insertion site. PEG, percutaneous endoscopic gastrostomy

Contrast-enhanced CT performed two days later confirmed dilated proximal small bowel with distal collapse and identified a transition point in the ileum (Figure 1). Oral contrast opacified the stomach but did not advance beyond it, indicating high-grade obstruction. The transition point lay immediately adjacent to the PEG insertion site, with surrounding fibrotic tissue changes. There was no free air, pneumatosis intestinalis, or evidence of bowel ischemia; mesenteric vessels were well opacified. No hernia or mass lesion was identified. After multidisciplinary discussion between gastroenterology, surgery, and internal medicine, a decision was made to proceed with surgical exploration. The nine-day interval between the diagnostic CT and surgery reflected the time required for optimization of the patient’s anticoagulation, given his antiphospholipid syndrome and recent therapeutic-dose enoxaparin, coordination between gastroenterology and surgical teams across two facilities, and assessment of his overall perioperative fitness in the context of concurrent critical limb ischemia. As there was no evidence of bowel ischemia, free perforation, or clinical deterioration during this period, a brief period of watchful waiting was considered clinically justified.

Surgical management

**Figure 2:**
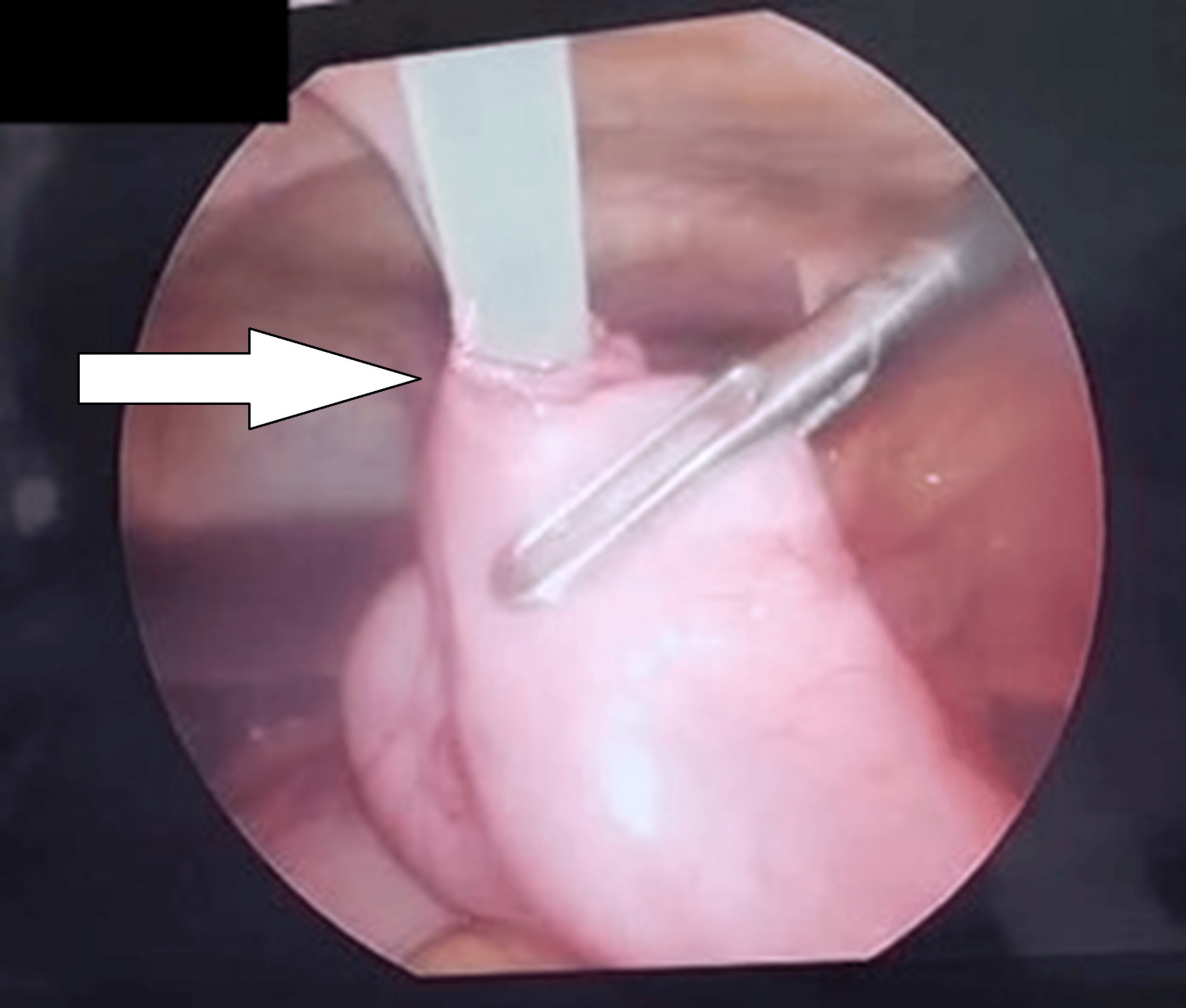
Intraoperative laparoscopic view showing the gastrostomy tube traversing the adherent loop of ileum at the site of a through-and-through perforation prior to release and primary repair.

Hybrid laparoscopic-endoscopic surgical exploration was carried out nine days after the diagnostic CT. A 5 mm Visiport was used for the initial port at the left palmar point following Veress needle insufflation, with three further 5 mm ports placed. At laparoscopic inspection, a loop of ileum was found firmly adherent to the anterior gastric wall at the PEG entry site; the gastrostomy tube had eroded straight through both walls of this loop, producing two full-thickness defects, an entry wound and an exit wound (Figure 2). No free intraperitoneal fluid was identified, and systematic review of the small bowel from the duodenojejunal flexure to the ileocecal junction revealed no additional abnormality.

The existing tube was divided flush with the skin, and the distal segment pushed into the peritoneal cavity. The adherent ileal loop was carefully freed from the anterior gastric wall. The tube was advanced back into the gastric lumen. Both ileal perforation sites were closed with 3-0 Vicryl interrupted pursestring sutures, and the gastric wall defect was closed with a continuous 3-0 Vicryl pursestring suture. The remnant tube was retrieved endoscopically, and a new gastrostomy was fashioned under combined laparoscopic and endoscopic guidance in the same operative sitting. The peritoneal cavity was irrigated with saline, a pelvic drain was placed, and all port sites were closed with 3-0 Vicryl.

Postoperative course

**Figure 3:**
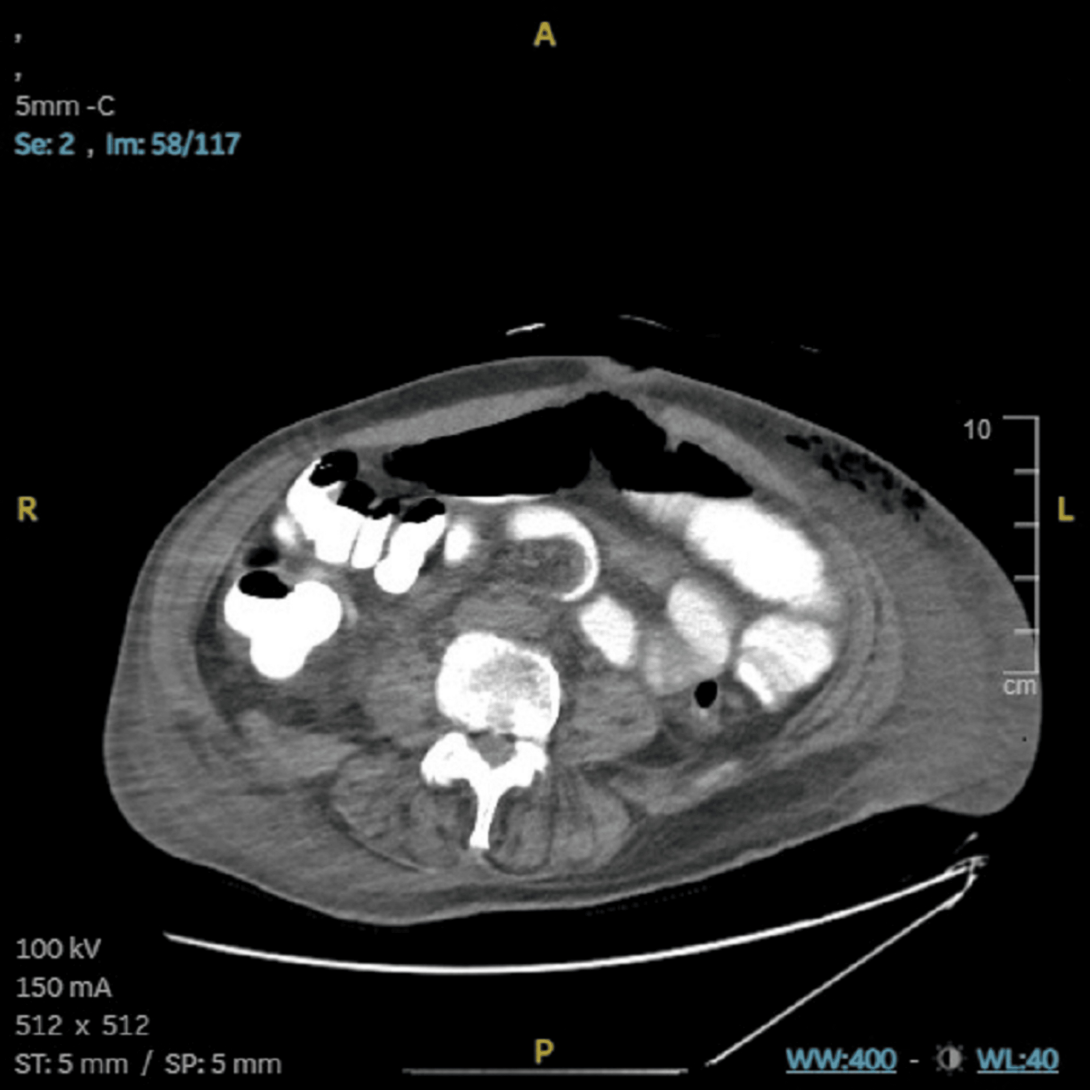
Follow-up contrast-enhanced CT obtained five days after surgical repair, demonstrating free passage of contrast through the bowel to the rectum, with complete resolution of the obstruction and the new gastric tube in a satisfactory position.

Follow-up contrast-enhanced CT performed five days postoperatively demonstrated free passage of oral contrast to the rectum with complete resolution of obstruction (Figure 3). The new gastric tube was in good position, with no dilated stomach, free air, or intraperitoneal collection. Bilateral moderate pleural effusions with basal atelectasis and a localized subcutaneous fluid collection with air loculi in the left flank were noted, attributed to localized postoperative inflammation, and managed conservatively. The patient subsequently tolerated enteral feeding via the revised gastrostomy without further vomiting or obstruction. Table 2 illustrates the whole timeline of the case presentation.

**Table 2:** Clinical timeline from admission to resolution of obstruction. PEG, percutaneous endoscopic gastrostomy

Time point	Event
2025	Admission: fever, confusion, poor oral intake; multidrug-resistant *Escherichia coli*UTI on culture
During admission	Treated for aspiration pneumonia, UTI, and COVID-19; acute left lower limb ischemia managed conservatively
Day 0	PEG tube insertion (20 Fr, pull technique); uncomplicated procedure
Day 1	Enteral feeding commenced at 10 mL/hr with planned advancement
Day 10	Recurrent vomiting and feed intolerance; plain CT shows small bowel obstruction
Day 12	Contrast CT: ileal transition point adjacent to PEG site with surrounding fibrotic change; multidisciplinary decision to operate
Day 21	Hybrid laparoscopic-endoscopic exploration: through-and-through ileal perforation by gastrostomy tube identified; bowel repaired; new gastrostomy fashioned in same sitting
Day 26	Follow-up contrast CT: complete resolution of obstruction; new tube in satisfactory position

## Discussion

The major complication rate for PEG insertion is consistently reported to be less than 5% in large series [2-11]. Small bowel obstruction directly attributable to the tube is much rarer, with the literature largely limited to isolated case reports [4-6]. Three principal mechanisms have been described: interposition of a bowel loop between the stomach and abdominal wall at insertion (the sandwich phenomenon) [3], internal bumper migration causing secondary obstruction (buried bumper syndrome) [5-8], and, as in our patient, progressive erosion of the tube through an adherent bowel loop, producing full-thickness perforation and mechanical obstruction. This third mechanism is likely the least commonly reported and may be the most diagnostically challenging, since symptoms develop insidiously days after an apparently uneventful procedure.

In this case, an ileal loop was probably lying in close proximity to the anterior gastric wall at the time of PEG insertion. Sustained pressure from the indwelling tube over 10 days appears to have caused progressive adhesion formation followed by full-thickness erosion through the bowel wall. Reviewing the published literature, true delayed ileal perforation by an indwelling PEG tube, as opposed to immediate intraoperative bowel injury, is exceptionally uncommon, with fewer than 10 cases identifiable in the English-language literature. Blumenstein et al. and Richter-Schrag et al. have catalogued the spectrum of PEG-related intra-abdominal injuries across large patient series, but delayed erosion through an adherent small bowel loop is not among the commonly tabulated complications in either registry [6-10]. The mechanism in our case, involving adhesion formation between the ileum and anterior gastric wall followed by progressive tube erosion over 10 days, differs from the classical intraoperative sandwich phenomenon, which typically presents within the first 24-72 hours as acute or early subacute obstruction [11]. Yamazaki et al. described a similar delayed presentation in a case of colo-cutaneous fistula after PEG, illustrating that transmural erosion by the tube can affect any adherent viscus depending on anatomical proximity at the time of insertion [11]. Our case shares features with those prior reports, such as insidious onset, initially nonspecific symptoms, and the critical role of contrast CT in establishing the diagnosis, but is distinguished by the bilateral through-and-through ileal perforation and the successful single-sitting hybrid repair.

Several mechanisms of PEG-related bowel injury have been proposed and deserve consideration in any such case. First, bowel interposition at insertion, the sandwich phenomenon, occurs when a loop of small or large bowel lies between the stomach and abdominal wall at the time of needle puncture, becoming incorporated into the tract [3]. This typically presents early and is more common when transillumination is inadequate or finger indentation is not used to confirm gastric wall apposition. Second, pressure necrosis from excessive external bumper tension is a well-recognized cause of buried bumper syndrome and has been proposed as a contributory mechanism in cases where the tube exerts sustained axial force against the gastric or abdominal wall [8]. In our patient, the tube was secured at 20 cm with no apparent excess tension at insertion, making isolated bumper pressure a less likely primary driver. Third, preexisting adhesions, which are plausible in any patient with prior abdominal inflammation, prior surgery, or prolonged recumbency, may predispose to abnormal bowel fixation adjacent to the stomach, creating the anatomical precondition for tube erosion [9]. Our patient’s prolonged bedridden state, history of repeated infections, and likely chronic peritoneal micro-inflammation from pressure ulceration may all have contributed to the ileal loop’s adherence to the anterior gastric wall. Fourth, repeated mechanical stress from tube manipulation during feeding cycles may have accelerated erosion once adhesion had formed. Taken together, the mechanism in this case was likely multifactorial: preexisting adhesion created the anatomical substrate, and sustained tube pressure completed the injury over 10 days. The contrast-enhanced CT findings were central to the preoperative diagnosis: the transition point was located precisely at the PEG entry site with surrounding fibrotic change, distinguishing this from other causes of early postoperative small bowel obstruction.

Current guidelines from the European Society of Gastrointestinal Endoscopy (ESGE) and the American Society for Gastrointestinal Endoscopy (ASGE) provide important context for both the procedure and its complications. The ESGE guideline on enteral feeding recommends PEG insertion using the pull technique with periprocedural antibiotic prophylaxis and emphasizes that adequate transillumination and finger indentation must be confirmed before needle puncture to minimize the risk of inadvertent bowel injury [14]. The ASGE guideline similarly recommends early cross-sectional imaging in any patient with post-procedural abdominal symptoms that do not resolve within 24-48 hours, a threshold that, if applied in our case, would have led to imaging on day 11, consistent with the clinical course [15]. Neither guideline specifically addresses delayed tube erosion as a complication category, reflecting the rarity of this mechanism and the absence of sufficient cases to generate evidence-based recommendations. Rahnemai-Azar et al. and Richter-Schrag et al. have both called for systematic complication registries to better characterize rare events such as delayed bowel erosion, and our case supports this recommendation [9,10].

Several practical points emerge from this case. First, new-onset vomiting or feed intolerance in a PEG-fed patient should prompt early cross-sectional imaging rather than reassurance, particularly in patients, like ours, who cannot self-report symptoms [7]. Second, contrast-enhanced CT is the investigation of choice, as it can localize the obstruction precisely in relation to the tube entry site. Third, a hybrid laparoscopic-endoscopic approach enabled both definitive bowel repair and simultaneous creation of a new feeding access in a single sitting, avoiding a return to theater and minimizing physiological insult in an already frail patient. Fourth, early multidisciplinary involvement was essential given the competing perioperative risks posed by antiphospholipid syndrome and critical limb ischemia in this patient, requiring close collaboration between gastroenterology, surgery, and internal medicine.

To our knowledge, delayed ileal perforation by an indwelling PEG tube after an otherwise uncomplicated insertion remains very rarely documented in the literature [6]. A limitation of any single case report is its inability to establish incidence; however, such reports serve an important function in alerting clinicians to mechanisms that may otherwise escape consideration.

The relationship between chronic disease burden and PEG-related complication risk deserves explicit consideration, particularly as PEG insertion is most commonly performed in patients who are already medically complex. Prior abdominal surgery is the most frequently cited predisposing factor for adhesion-related visceral injury in the PEG literature, as post-surgical adhesions can fix bowel loops in abnormal proximity to the anterior gastric wall prior to tube placement [9,10]. However, our case illustrates that a clinically equivalent anatomical substrate can develop through entirely nonsurgical mechanisms in patients with significant chronic disease. Prolonged immobility, recurrent systemic infection, chronic pressure ulceration, and the low-grade peritoneal micro-inflammation associated with these conditions may collectively promote fibrinous adhesion formation between visceral surfaces without any antecedent surgical trigger [9]. In neurologically impaired, bedridden patients, who represent a substantial proportion of all PEG recipients, this risk is compounded by the inability to self-report early symptoms, meaning that complications may progress further before clinical recognition than they would in ambulant patients. Our patient exemplifies this: 10 days elapsed between tube insertion and clinical presentation, during which the adhesion matured and the tube eroded progressively through the ileal wall without any symptom the patient could communicate. This has practical implications for post-PEG monitoring protocols: clinicians should maintain a lower threshold for imaging in patients with significant chronic disease burden, prolonged recumbency, or active systemic infection at the time of tube insertion, even when the procedure itself appears uncomplicated [9,14,15].

## Conclusions

PEG tube-related ileal obstruction is rare, but it should be considered in any patient who develops vomiting or feed intolerance following gastrostomy tube insertion and does not settle promptly. Contrast-enhanced CT identifying the transition point in relation to the tube site, combined with early multidisciplinary surgical involvement, are the key factors enabling timely diagnosis and a successful outcome, as demonstrated in this case. In our patient, the hybrid laparoscopic-endoscopic approach allowed definitive repair of the bowel injury and simultaneous creation of a new feeding access in a single operative sitting, avoiding a return to theater and enabling early resumption of enteral nutrition within five days of surgery, an outcome that would have been considerably harder to achieve in this frail, high-risk patient with a staged approach. This case adds to the very limited literature on delayed tube erosion as a mechanism of PEG-related small bowel obstruction. It reinforces that awareness of this complication, even after an apparently uncomplicated procedure, can prevent diagnostic delay and its associated morbidity.
